# Does treatment strategy influence the ability to achieve and sustain DMARD-free remission in patients with RA? Results of an observational study comparing an intensified DAS-steered treatment strategy with treat to target in routine care

**DOI:** 10.1186/s13075-019-1893-z

**Published:** 2019-05-07

**Authors:** L. E. Burgers, J. A. van der Pol, T. W. J. Huizinga, C. F. Allaart, A. H. M. van der Helm-van Mil

**Affiliations:** 1 Department of Rheumatology, Leiden University Medical Center, C-01-046, PO Box 9600, 2300 RC Leiden, the Netherlands; 2 Department of Rheumatology, Erasmus Medical Center, Rotterdam, the Netherlands

**Keywords:** Rheumatoid arthritis, Epidemiology, Outcome measures, DMARDs, Study design

## Abstract

**Objectives:**

To study the impact of treatment strategy on achieving and sustaining disease-modifying antirheumatic drug (DMARD)-free remission in patients with rheumatoid arthritis (RA).

**Methods:**

Two hundred seventy-nine RA patients (median follow-up 7.8 years) were studied. Of these, 155 patients participated in a disease activity score (DAS) < 1.6 steered trial aimed at DMARD-free remission. Initial treatment comprised methotrexate with high-dose prednisone (60 mg/day) and a possibility to start biologicals after 4 months. In the same period and hospital, 124 patients were treated according to routine care, comprising DAS < 2.4 steered treatment. Percentages of DMARD-free remission (absence of synovitis for ≥ 1 year after DMARD cessation), late flares (recurrence of clinical synovitis ≥ 1 year after DMARD cessation), and DMARD-free *sustained* remission (DMARD-free remission sustained during complete follow-up) were compared between both treatment strategies.

**Results:**

Patients receiving intensive treatment were younger and more often ACPA-positive. On a group level, there was no significant association between intensive treatment and DMARD-free remission (35% vs 29%, corrected hazard ratio (HR) 1.4, 95%CI 0.9–2.2), nor in ACPA-negative RA (49% versus 44%). In ACPA-positive RA intensive treatment resulted in more DMARD-free remission (25% vs 6%, corrected HR 4.9, 95%CI 1.4–17). Intensive treatment was associated with more late flares (20% versus 8%, HR 2.3, 95%CI 0.6–8.3). Subsequently, there was no difference in DMARD-free *sustained* remission on a group level (28% versus 27%), nor in the ACPA-negative (43% versus 42%) or ACPA-positive stratum (17% versus 6%, corrected HR 3.1, 95%CI 0.9–11).

**Conclusions:**

Intensive treatment did not result in more DMARD-free sustained remission, compared to routine up-to-date care. The data showed a tendency towards an effect of intensive treatment in ACPA-positive RA; this needs further investigation.

**Electronic supplementary material:**

The online version of this article (10.1186/s13075-019-1893-z) contains supplementary material, which is available to authorized users.

## Introduction

Over the last decades, treatment of rheumatoid arthritis (RA) has changed dramatically. Treatment targets have shifted from mere relief of symptoms towards treat-to-target therapy aimed at remission and prevention of structural joint damage [1–3]. The recent European League Against Rheumatism (EULAR) recommendations for the management of RA state that treatment should be aimed at sustained remission or low disease activity, defined according to Boolean or index-based definitions, which correspond with the absence of radiologic damage [2, 4]. These treatment aims can be achieved while patients are still on disease-modifying antirheumatic drugs (DMARDs).

Although RA is considered a chronic disease, there is growing evidence that a proportion of patients can achieve DMARD-free remission with reported percentages ranging between 3.6 and 23% [5–12]. To note, varying definitions of DMARD-free remission were used in these studies. DMARD-free sustained remission, which has been defined as the sustained absence of arthritis after cessation of DMARDs, may be interpreted as the closest proxy to cure of RA, especially as it also corresponds with a patient-perceived state of remission in terms of normalized levels of physical functioning, pain, fatigue, and stiffness [13, 14]. Although studies have shown it is an achievable goal in part of RA patients, EULAR recommendations are cautious with regard to tapering and stopping DMARDs. The main reason for this being the lack of evidence about safely stopping DMARD therapy and the risk of flares [2, 15–17].

The presence of RA-related autoantibodies associates with a decreased risk of DMARD-free sustained remission [10, 13], but biologic mechanisms mediating resolution of RA chronicity are mostly unknown [5]. Studies have shown that with better treatment options and the introduction of disease activity score (DAS)-steered treatment, DMARD-free remission has become a more achievable outcome [13]. However, it is unclear if current DAS-steered treatment, starting with methotrexate (MTX), results in an optimum chance for achieving this outcome, or whether a more intensive DAS-steered treatment regimen can result in an even higher proportion of patients achieving and sustaining DMARD-free remission.

Therefore, this study assessed if treatment strategy impacts the chance of disease resolution. We compared the prevalence of DMARD-free remission, as well as DMARD-free sustained remission between patients treated according to an intensive DAS-steered treatment strategy as applied in the setting of a clinical trial (the IMPROVED study) [18] and patients treated according to routine care, in line with EULAR recommendations [2]. All studied patients were treated in the same center by the same rheumatologists. In short, trial patients were treated DAS (< 1.6) steered and started with high-dose prednisone next to MTX, whereas routine care consisted of initial MTX- and DAS (< 2.4) steered treatment.

## Methods

### Patients

**Fig. 1:**
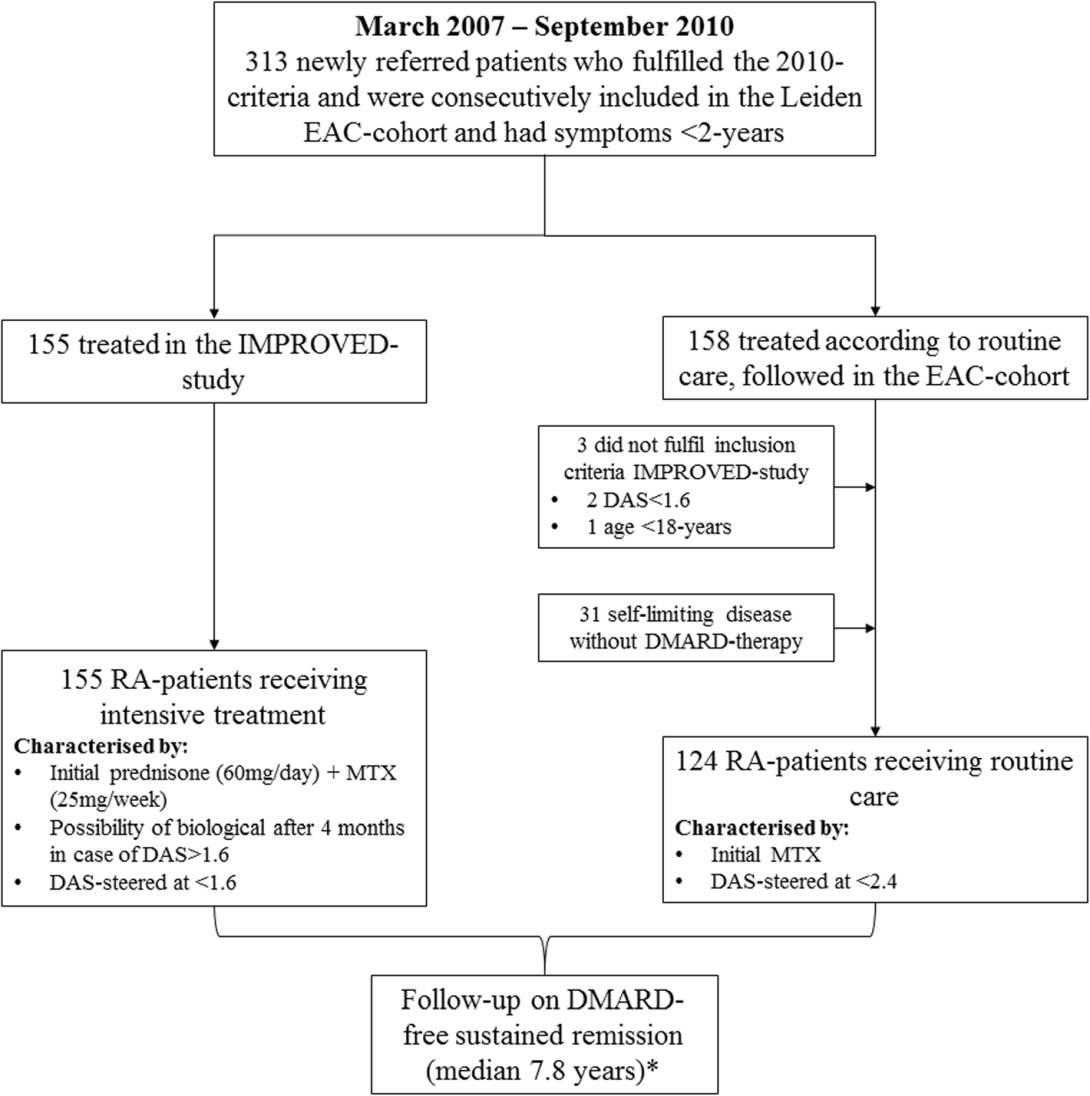
Flowchart of patient selection. Figure depicting patient selection for the current study. EAC, early arthritis clinic; RA, rheumatoid arthritis; DAS, disease activity score; DMARD, disease-modifying antirheumatic drug; MTX, methotrexate. Patients in the regular treatment group that did not fulfill the inclusion criteria of the IMPROVED study were excluded for reasons of comparability. *For patients who participated in the IMPROVED study, follow-up after conclusion of the study (after 5 years) continued in the EAC cohort according to routine care

All patients who were newly diagnosed with RA (according to the 2010 criteria) between March 2007 and September 2010 in the Leiden University Medical Center *and* who were included in the Leiden Early Arthritis Clinic (EAC) [19] were selected for this study (*n* = 313, Fig. 1). The EAC is a prospective, population-based inception cohort that includes patients with clinically confirmed arthritis and a symptom duration < 2 years [19]. Besides regular visits with their rheumatologist, patients had scheduled study visits at least once a year, including questionnaires, physical examination, and blood samples. All patients were treated by the same team of rheumatologists in the same center, but according to different treatment strategies; either according to an intensive DAS-steered treatment regimen within the IMPROVED study [18] or according to up-to-date routine care (see below for more details). In order to study a homogenous group of patients, those who did not fulfill the inclusion criteria of the IMPROVED study (see below) or were not started on DMARD therapy were excluded (*n* = 34) (Fig. 1). Thus, a total of 279 patients were studied. In principle, all patients could have been included in the IMPROVED study. Nevertheless, only 155 patients were included. Reasons why 124 patients were not included were not routinely documented, but could either be patient related (for example, patient did not want to participate), rheumatologist related (rheumatologist did not ask patient to participate), or both.

### Intensive treatment

The IMPROVED study is a multicenter randomized single-blinded clinical trial that recruited 610 patients between March 2007 and September 2010 [18]. For inclusion, patients had to be ≥ 18 years, have a diagnosis of early RA or UA, a DAS ≥ 1.6, and no prior use of DMARDs. In contrast to routine care, all patients were started on high-dose prednisone 60 mg/day which was tapered to 7.5 mg/day in 7 weeks and MTX, starting at 7.5 mg per week and escalated to 25 mg/week. If patients were in early remission (DAS < 1.6) after 4 months, prednisone was tapered to stop and if patients were still in remission after 8-months MTX was tapered and stopped as well over the next 4 months. If patients were not in remission after 4 months, they were randomized either to adding hydroxychloroquine and sulphasalazine to MTX and prednisone or to switching to MTX plus adalimumab. Patients had 4 monthly visits, and medication was tapered or stopped in case of a DAS < 1.6 and restarted, switched, or increased in case of a DAS ≥ 1.6. Primary outcomes were DAS-remission and drug-free remission based on a DAS < 1.6. The study has previously been described [18, 20] and was approved by the Medical Ethical Committee. All patients provided written informed consent. For the present study, only patients included in the Leiden University Medical Center and who fulfilled the 2010 criteria for RA were studied (*n* = 155). Thus, this concerned a subgroup of patients from the total IMPROVED study (Fig. 1). The follow-up duration of the IMPROVED study was 5 years; thereafter, follow-up of all patients continued within the EAC cohort and patients were treated routinely.

### Routine care

In line with EULAR recommendations [2] and local guidelines, routine care comprised DAS (< 2.4) steered treatment, where treatment was initially started with MTX. In case of a DAS < 2.4, treatment was generally tapered and eventually stopped, whereas in case of a DAS ≥ 2.4, treatment was intensified. After the failure of > 2 conventional DMARDs, biologics were allowed. Thus, similar to patients receiving intensive treatment, routine care was DAS steered and tapering and stopping of DMARDs was routine; however, the treatment target differed (DAS < 1.6 versus DAS < 2.4). Of all patients receiving routine care, 67 (54%) received initial combination therapy of MTX with corticosteroids (either oral prednisone (median starting dose 10 mg/day (interquartile range 7.5, 17.5) or intramuscular as Methylprednisolone with doses ranging between 80 and 120 mg). During complete follow-up, 17 patients (14%) were treated with biologicals, but none were initiated within the first year of follow-up. The course of the DAS score in patients receiving routine care is depicted in Additional file 1: Figure S1.

Ethical approval. The study was approved by the Medical Ethical Committee of the LUMC (“Commissie Medische Ethiek LUMC”). All patients provided written informed consent.

### Outcome

Three outcomes were studied. The first was DMARD-free remission, defined as the absence of clinical synovitis for ≥ 1 year after DMARD cessation; hence, patients with early recurrence of synovitis after DMARD stop were not included in this group. The second outcome was the occurrence of late flares, defined as recurrence of clinical synovitis *after* having achieved DMARD-free remission: thus, a recurrence of clinical synovitis more than 1 year after DMARD cessation. So, if patients had a recurrence of clinical synovitis within 1 year after DMARD cessation, this was not considered a late flare; then, follow-up continued and patients were considered as not being in DMARD-free remission. Thirdly, DMARD-free *sustained* remission, defined as the absence of clinical synovitis for > 1 year after DMARD cessation *and* for the remainder of the follow-up, was studied. Thus, these were the patients in DMARD-free remission minus those with a late flare. Importantly, these outcomes were different from the outcomes studied in the IMPROVED study, which were sustained drug-free remission, defined as a period of drug-free remission based on a DAS < 1.6 for ≥ 1 year, *regardless* of a need to restart DMARD therapy after this period, radiographic joint damage, and functional disability. Radiographic damage was not studied here as, based on previous studies including the IMPROVED study, we expected little clinically relevant joint damage [18, 21]. All medical records were assessed on these outcomes between March and May 2017. If patients were in DMARD-free (sustained) remission, the date of remission was the date 1 year after cessation of DMARDs. For patients not in DMARD-free (sustained) remission, the censoring date was either the date of going through the medical records, or an earlier date in case patients was lost to follow-up or had died.

### Statistics

Baseline characteristics were compared using Students’ *t* tests, chi-square tests, and Mann-Whitney *U* tests, as appropriate. Kaplan-Meier curves were used to depict the occurrence of DMARD-free (sustained) remission and late flares over time. Univariable and multivariable Cox-regression proportional hazards models were used to study associations between treatment strategy and achieving DMARD-free (sustained) remission or late flares. Multivariable models were adjusted for baseline differences. An analysis corrected for the propensity score was performed as a sensitivity analysis to reduce possible bias caused by confounding by indication (Additional file 1: Supplementary methods). Because a baseline difference in anticitrullinated protein antibody (ACPA) positivity was observed and ACPA positivity is associated with a lower risk of DMARD-free remission [10, 13], analyses were stratified by ACPA status (EliA CCP2, Phadia, Nieuwegein, the Netherlands, positive if ≥ 7 U/mL, determined at baseline). Analyses were performed in SPSS version 24.0. *P* values < 0.05 were considered statistically significant.

## Results

### Baseline characteristics

Baseline characteristics are depicted in Table 1. Most characteristics were similar for both treatment strategies, but patients receiving intensive treatment were younger (mean of 53 versus 61 years, *p* < 0.001) and more often auto-antibody positive (59% versus 40% for ACPA positivity, *p* = 0.003 and 65% versus 48% for rheumatoid factor (RF) positivity, *p* = 0.005). The median follow-up duration of all patients was 7.8 years (IQR 6.8–8.7 years) and was similar for both treatment strategies (median 7.8 years in both groups).

**Table 1:** Baseline characteristics

	RA patients receiving intensified treatment (*n* = 155)	RA patients receiving routine care (*n* = 124)	*p* value
Age, mean (SD)	53 (14)	61 (15)	< 0.001
Female gender, *n* (%)	106 (68)	80 (65)	0.50
Symptom duration < 12 weeks, *n* (%)	60 (39)	47 (38)	0.81
66-SJC, median (IQR)	6 (3–11)	7 (3–11)	0.79
68-TJC, median (IQR)	12 (7–20)	12 (6–19)	0.32
ESR, median (IQR)	25 (11–41)	31 (14–46)	0.25
DAS44, median (IQR)	3.1 (2.6–3.8)	3.0 (2.5–3.7)	0.30
ACPA-positive, *n* (%)*	92 (59)	50 (40)	0.003
RF-positive, *n* (%)*	101 (65)	60 (48)	0.005

Table depicting baseline characteristics of all patients included in the present study. Missings were as follows: ACPA (2), TJC (9), SJC (8), ESR (1), DAS44 (11). *ACPA-positive if ≥ 7 U/mL and IgM rheumatoid factor (RF)-positive if ≥ 3.5 IU/mL. *RA* rheumatoid arthritis, *SD* standard deviation, *SJC* swollen joint count, *TJC* tender joint count, *IQR* interquartile range, *ESR* erythrocyte sedimentation rate, *ACPA* anticitrullinated protein antibody, *RF* rheumatoid factor

### DMARD-free remission

**Fig. 2:**
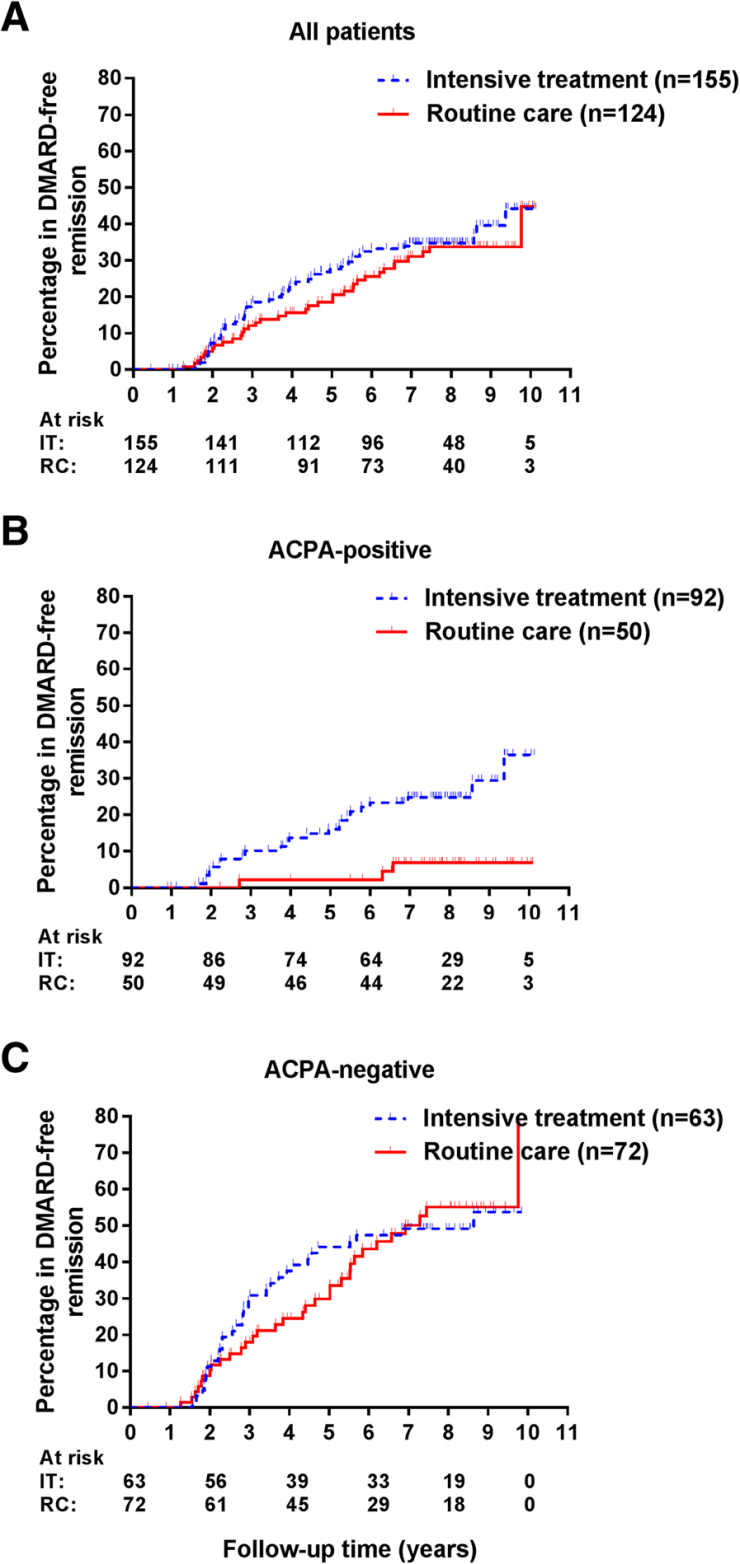
Kaplan-Meier curves depicting the percentage of patients achieving DMARD-free remission by treatment strategy. Figure depicting comparisons of DMARD-free remission by treatment strategy in all included patients (**a**) and stratified by ACPA status (**b, c**). DMARD, disease-modifying antirheumatic drug; HR, hazard ratio; ACPA, anticitrullinated protein antibodies; IT, intensive treatment; RC, routine care

DMARD-free remission was achieved by 35% (54/155) of patients receiving intensive treatment after a median of 3.0 years and by 29% (36/124) of patients receiving routine care after a median of 4.1 years (Fig. 2a). Baseline characteristics of these 54 and 36 patients are shown in Additional file 1: Table S1 and reveal that patients achieving DMARD-free remission in the intensive treatment arm were older and more often auto-antibody positive compared to routine care (thus, similar to the baseline characteristics on group level). An intensive treatment strategy was not significantly associated with a higher chance on achieving DMARD-free remission (hazard ratio (HR) 1.2, 95%CI 0.8–1.8). Also, after correction for baseline differences (age, ACPA, and RF), no significant association was observed (HR 1.4, 95%CI 0.9–2.2).

Because of the baseline difference in ACPA positivity between the groups and because we hypothesized that an impact of treatment strategy on DMARD-free remission might be different in ACPA-positive and ACPA-negative RA patients, analyses were stratified by ACPA status. Within both strata, patient characteristics were similar, except for a younger age in patients receiving intensive treatment in both strata (Table 2). Within ACPA-positive patients, intensive treatment was associated with a higher chance on achieving DMARD-free remission (25% versus 6%, HR corrected for age 4.9, 95%CI 1.4–16.9, Fig. 2b). Within ACPA-negative RA patients, the percentage of patients achieving DMARD-free remission was similar for both treatment strategies (49% versus 44% in routine care, Fig. 2c). No Cox-regression analysis was performed within ACPA-negative patients as the assumption of proportional hazards was not met (Fig. 2c).

**Table 2:** Baseline characteristics stratified for the presence of ACPA

	ACPA-positive		*p* value	ACPA-negative		*p* value
	RA patients receiving intensified treatment (*n* = 92)	RA patients receiving routine care (*n* = 50)		RA patients receiving intensified treatment (*n* = 63)	RA patients receiving routine care (*n* = 72)	
Age, mean (SD)	50 (12)	58 (12)	*0.001*	56 (16)	63 (16)	*0.019*
Female gender, *n* (%)	66 (72)	32 (64)	0.34	40 (64)	47 (65)	0.83
Symptom duration < 12 weeks, *n* (%)	31 (36)	12 (27)	0.31	25 (42)	28 (44)	0.82
66-SJC, median (IQR)	5 (3–10)	6 (3–8)	0.73	8 (4–15)	8 (4–14)	0.96
68-TJC, median (IQR)	11 (6–16)	8 (4–16)	0.06	16 (11–25)	14 (10–21)	0.34
ESR, median (IQR)	29 (16–41)	30 (14–44)	0.60	19 (9–41)	31 (11–52)	0.19
DAS44, median (IQR)	2.9 (2.5–3.5)	2.7 (2.4–3.1)	0.21	3.3 (2.7–4.0)	3.2 (2.6–3.8)	0.66
RF-positive, *n* (%)	81 (88)	40 (80)	0.20	20 (32)	18 (25)	0.39

Table depicting baseline characteristics of all patients included in the present study, stratified for ACPA status. Missings were as follows: ACPA (2), TJC (9), SJC (8), ESR (1). *RA* rheumatoid arthritis, *SD* standard deviation, *SJC* swollen joint count, *TJC* tender joint count, *IQR* interquartile range, *ESR* erythrocyte sedimentation rate, *ACPA* anticitrullinated protein antibody, *RF* rheumatoid factor

### Late flares

**Fig. 3:**
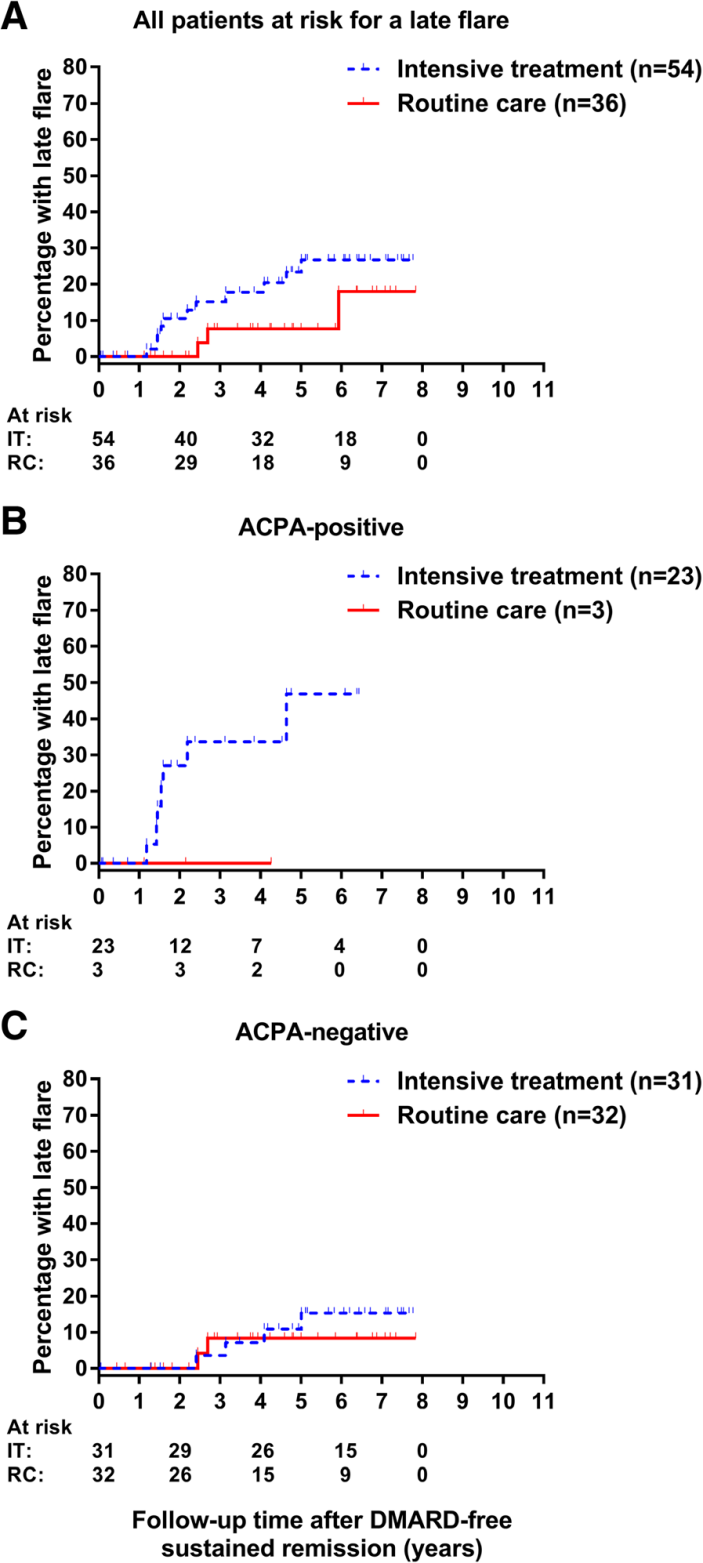
Kaplan-Meier curve depicting the occurrence of late flares by treatment strategy. Kaplan-Meier curve depicting the occurrence of late flares by treatment strategy in patients who achieved DMARD-free remission (*n* = 90) in the whole group (**a**) and stratified by ACPA-status (**b**, **c**). DMARD, disease-modifying antirheumatic drug; IT, intensive treatment; RC, routine care

A total of 90 patients achieved DMARD-free remission, and these patients were at risk for having a late flare. In these patients, the median follow-up after having achieved DMARD-free remission was 4.7 (95%CI 2.2–6.4) years. A late flare was observed in 20% (11/54) of patients receiving intensive treatment and in 8% (3/36) of patients in the routine care group (HR 2.3, 95%CI 0.6–8.3, Fig. 3a–c). No multivariable analyses or stratified analyses were performed, because of the small number of events. Of the 14 patients in whom a late flare occurred, 8 presented with polyarthritis at the time of flare, 4 with oligoarthritis, and 2 with monoarthritis. The presence of ACPA and/or RF was associated with the occurrence of a late flare (Additional file 1: Table S2; HR 1.23 (1.02–1.50) for ACPA and 6.34 (1.77–22.79) for RF positivity).

### DMARD-free sustained remission

**Fig. 4:**
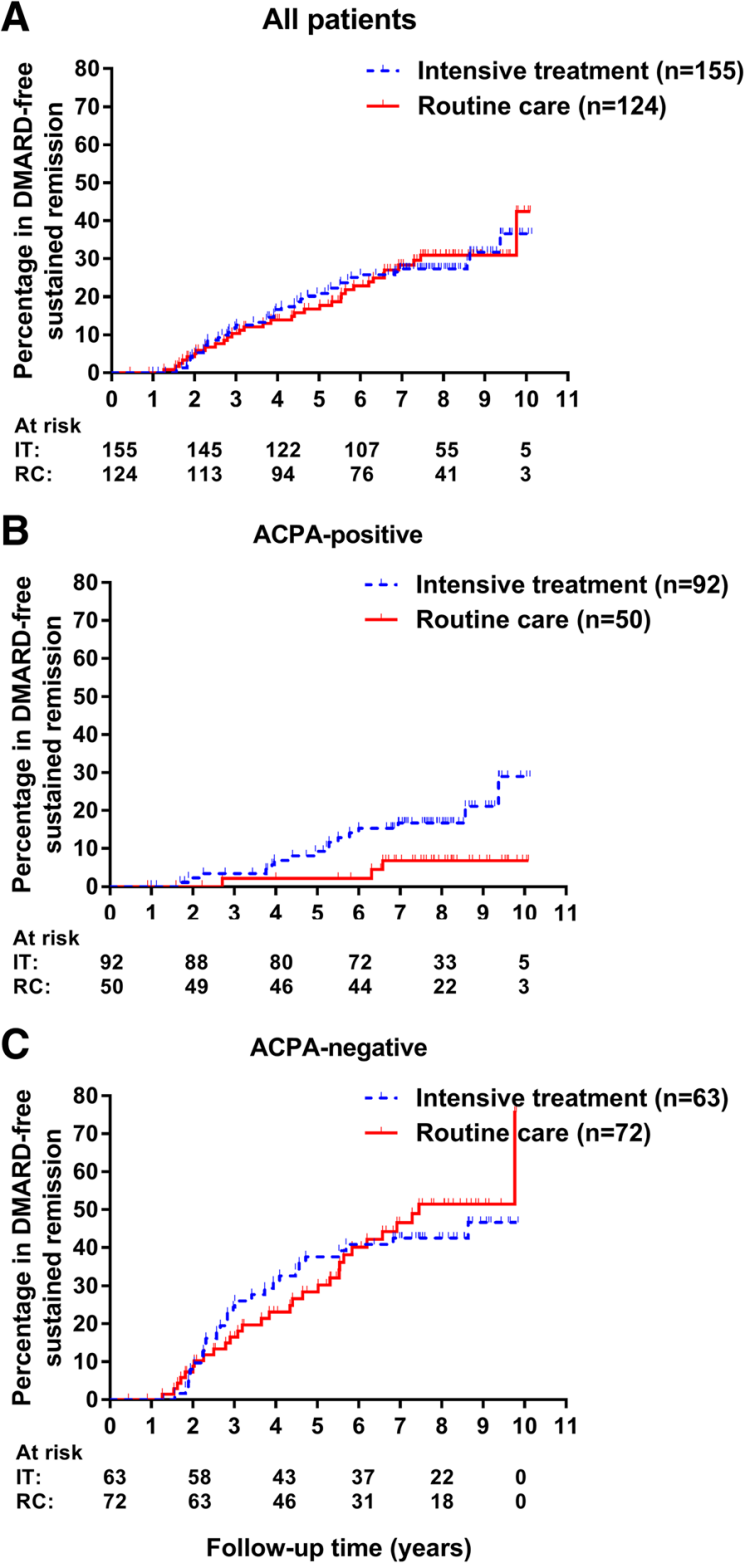
Kaplan-Meier curves depicting the percentage of patients achieving DMARD-free *sustained* remission by treatment strategy. Kaplan-Meier curves depicting the achievement of DMARD-free sustained remission by treatment strategy in all included patients (**a**) and stratified by ACPA-status (**b**, **c**). DMARD, disease-modifying antirheumatic drug; HR, hazard ratio; ACPA, anticitrullinated protein antibodies; RF, rheumatoid factor; IT, intensive treatment; RC, routine care

Patients with late flares were not included in the group of RA patients that achieved DMARD-free sustained remission. DMARD-free remission that sustained until the end of follow-up was observed in 28% (43/155) of patients receiving intensive treatment and in 27% (33/124) of patients receiving routine care. Also here, the assumption of proportional hazards was not met; thus, Cox-regression analyses were not performed. However, these data showed no difference between the groups (Fig. 4a).

Within the ACPA-positive stratum, DMARD-free sustained remission was achieved by 17% (16/92) of patients receiving intensive treatment and by 6% (3/50) of patients receiving routine care (Fig. 4b). This difference was not statistically significant (HR corrected for age 3.1, 95%CI 0.9–11.0). Within the ACPA-negative stratum, the percentage of patients achieving DMARD-free sustained remission was similar (43% versus 42% in routine care, Fig. 4c).

In order to further decrease, the chance on confounding by indication, analyses on DMARD-free sustained remission were repeated and corrected for the propensity score. This revealed similar results, namely that patients receiving intensive treatment had a HR of 1.3 (95%CI 0.8, 2.1) on achieving DMARD-free sustained remission.

## Discussion

This study compared a treatment strategy that is intensive in treatment target and in the medications used with an up-to-date regular treatment strategy, with DMARD-free sustained remission as long-term outcome. An intensive treatment strategy was not associated with a higher prevalence of DMARD-free sustained remission, nor after correction for baseline differences, nor after correction for a propensity score. Stratification for ACPA revealed that ACPA-positive RA patients in the intensive treatment group achieved DMARD-free remission more often but also had a higher rate of late flares. Therefore, our current results do not provide evidence for long-term benefits of an intensive DAS-steered treatment regimen with regard to achieving DMARD-free sustained remission.

While a randomized trial would have been the best method to compare the two treatment strategies because of the element of randomization, the present study does have some important advantages. The first is the long-term follow-up duration. Trials often have a limited follow-up duration which hampers the evaluation of long-term outcomes. In the IMPROVED trial, the follow-up was restricted to 5 years. The median follow-up in this study was almost 8 years as patients were followed-up in the EAC cohort after conclusion of the trial, and some patients had a follow-up of > 10 years.

A second strength of our study is that the whole source population of RA patients newly classified with RA in a time-period in one center was studied. Trials include sets of patients with certain characteristics, hampering extrapolation to the general population of RA-patients. Furthermore, the IMPROVED trial did not include a regular treatment arm [18]. Comparing RA patients treated in this trial with RA patients treated by the same team of rheumatologists according to routine care allowed to evaluate whether an intensive trial regimen is favorable for the long-term outcome studied. As mentioned previously, an important issue is why almost half of the recent-onset RA patients that met the inclusion criteria of the IMPROVED study did not participate. Reasons for not participating were not routinely documented and may be related to willingness of the patient or preference of the rheumatologist. Relatively few differences in patient characteristics were observed between the two groups. The most important difference was the prevalence of ACPA; possibly rheumatologists or patients themselves were less motivated in case of ACPA negativity. Since ACPA has been associated with a lower hazard on achieving DMARD-free remission [10, 13], analyses were repeated after stratification by ACPA status to prevent bias. In the whole group, multivariable models corrected for baseline differences as well as for a propensity score were performed in order to reduce bias caused by confounding by indication. Altogether, the issue of non-comparability might not be completely prevented in this way. However, although there may still be unmeasured confounding, there are also many important similarities between the two groups. These include similarities in patient characteristics, inclusion period, center, and team of treating rheumatologists. This suggests that the differences observed could be largely attributed to the most important difference between the groups, namely the treatment strategy that was applied.

The outcome DMARD-free sustained remission is infrequently studied. A previous study from our center evaluated the difference in DMARD-free remission during 5 years of follow-up in patients with DAS-driven versus non-DAS-driven therapy [22]. Here, the DAS-driven group was derived from the BeSt-trial [21]. Because of stringent inclusion criteria, a rather severe set of RA patients was included in the trial. As shown by the many differences in baseline characteristics between both treatment groups [22], non-comparability was a larger issue in this previous study than in the present investigation.

Despite some methodological limitations, data from this previous study suggested that ACPA-positive patients had a greater advantage of DAS-driven therapy [22]. Also, in our data, ACPA-positive patients achieved DMARD-free remission more often in the intensive therapy group. However, after considering the late flares, there was no significant difference in the DMARD-free remission that was sustained over time. It is possible that the remaining difference would have reached statistical significance if the sample size would have been larger. In contrast, late flares occurred more often in ACPA-positive patients after having achieved DMARD-free remission. Consequently, it is also possible that after a longer follow-up, the rate of late flares would increase especially in the ACPA-positive group, diminishing the difference between the intensive treatment group and routine care in ACPA-positive RA. Hence, the present data do not allow to conclude that ACPA-positive RA patients benefit from an intensive treatment strategy with regard to achieving and sustaining DMARD-free remission.

Although the long follow-up duration is advantageous and allowed to study the occurrence of late flares, some late flares occurred several years after DMARD cessation. Possibly, the currently observed percentage of patients achieving late flares is underestimated. In addition, some late flares may have been missed as patients in sustained remission can be referred to the GP with instructions to return if symptoms reoccur. Despite these instructions and the fact that early access for RA patients is promoted in several ways, including the presence of screening clinics [23], we cannot exclude that some patients were not referred back to our outpatient clinic in case of recurring symptoms. However, we do not expect that these issues, if present, depend on treatment strategy or ACPA status. Similarly, the amount of patients achieving DMARD-free *sustained* remission could be either an underestimation (as more patients may achieve this after longer follow-up) or an overestimation (as patients already having achieved this outcome could experience a recurrence of clinical synovitis). However, as follow-up duration was similar in both treatment arms, we do not expect this would change the results.

Differences in treatment strategy between the two groups were not only the difference in treatment target (DAS < 1.6 instead of 2.4), but also the initial high dose of prednisone (60 mg/day), and the possibility to switch to biologicals after 4 months. In regular care, prednisone was occasionally started next to MTX, but not in a high dose and biologics were only allowed after failure of > 2 cDMARDS, which (if necessary) generally took place at a longer disease duration. In contrast to the patients treated in the trial, treatment changes in the regular care group were also made at non-protocolized visits and a larger variety of DMARDs were possible. This hampered a detailed registration of all DMARDs used in the regular care group. Additionally, it is possible that in routine care, rheumatologists were more reluctant to taper and stop DMARDs. However, despite all the differences in treatment strategy between the groups, no important differences in DMARD-free sustained remission were observed.

The IMPROVED study had a duration of 5 years; thereafter, patients were treated according to the best insights of the treating rheumatologist. Thus, after 5 years of treatment, the strategies became similar between both groups. This may have resulted in a reduction of initial contrasts between the groups [24].

The percentage of patients achieving DMARD-free sustained remission was relatively high, but similar to previous studies on this outcome [13]. Local treatment guidelines comprise tapering and stopping of DMARDs also in regular care [13]. This may differ from routine care elsewhere, especially since EULAR guidelines are cautious with regard to tapering and subsequent stopping of DMARDs [2, 16]. Now, several studies have revealed that DMARD-free remission is an achievable outcome; more research on tapering and stopping DMARDs is warranted.

## Conclusions

In conclusion, the present data showed no benefit from an intensive treatment regimen compared to routine care for the long-term outcome DMARD-free sustained remission in RA. Validation in a randomized setting is required. Based on the present results, an eventual benefit of an intensive treatment regimen is most expected in ACPA-positive RA. Because of the occurrence of late flares particularly in this group, a possible future trial on this proxy of cure of RA should have a follow-up duration of ≥ 10 years to come to definite conclusions.

## Additional file

Additional file 1: Supplementary methods. **Figure S1.** DAS44 over time. **Table S1.** Baseline characteristics of patients who achieved DMARD-free remission, stratified by treatment arm. **Table S2.** Univariable Cox-regression analyses studying the association between baseline characteristics and late flares in patients at risk for a late flare. (DOCX 79 kb)

## Abbreviations

ACPA: Anticitrullinated protein antibodies
CI: Confidence intervals
DAS: Disease activity score
DMARD: Disease-modifying antirheumatic drug
EAC: Early arthritis clinic
EULAR: European League Against Rheumatism
HR: Hazard ratio
MTX: Methotrexate
RA: Rheumatoid arthritis
RF: Rheumatoid factor
