# Supplementary material for: Does treatment strategy influence the ability to achieve and sustain DMARD-free remission in patients with RA? Results of an observational study comparing an intensified DAS-steered treatment strategy with treat to target in routine care
Source: Arthritis Res Ther. 2019 May 7;21:115. doi: 10.1186/s13075-019-1893-z (PMC6505077; doi:10.1186/s13075-019-1893-z)
Supplement: Supplementary file 1 — Supplementary methods. Figure S1. DAS44 over time. Table S1. Baseline characteristics of patients who achieved DMARD-free remission, stratified by treatment arm. Table S2. Univariable Cox-regression analyses studying the association between baseline characteristics and late flares in patients at risk for a late flare. (DOCX 79 kb) [file 13075_2019_1893_MOESM1_ESM.docx]

**Supplementary methods**

*Propensity score*

Our study is an observational study and thus patients were not assigned to a treatment strategy by randomization. Therefore, confounding by indication may have occurred, resulting in differences in baseline characteristics that may influence the studied associations between treatment strategy and DMARD-free (sustained) remission. To reduce possible bias a propensity score was calculated for each patient, representing the probability to be assigned to intensive treatment[1]. Variables that were selected for the model were those that could (in theory) be related both to assigning patients to a treatment arm and the outcome. To this end, baseline variables that were related to the outcome (DMARD-free sustained remission) were selected. Cox-regression was used to study the relation between baseline variables and DMARD-free sustained remission. Variables with a p<0.10 were used in the propensity score model (Age, symptom duration, RF and ACPA)[2]. Probit regression was used to come to a final balanced model, resulting in one final propensity score per patient. This score was then added as a covariate in the cox-regression analysis looking at the association between treatment strategy and achieving DMARD-free sustained remission (Supplementary table 2).

**REFERENCES**

1. Groenwold RHH. ‘Propensity scores’ in observationeel onderzoek. Ned Tijdschr Geneeskd [Internet]. 2013 Jul 9 [cited 2018 Feb 12]; Available from: https://www.ntvg.nl/artikelen/%E2%80%98propensity-scores%E2%80%99-observationeel-onderzoek

2. Spreeuwenberg MD, Bartak A, Croon MA, Hagenaars JA, Busschbach JJV, Andrea H, et al. The multiple propensity score as control for bias in the comparison of more than two treatment arms: an introduction from a case study in mental health. Med Care. 2010 Feb;48(2):166–74.

**Table S1: Baseline characteristics of patients who achieved DMARD-free remission, stratified by treatment arm**

|  | **Intensified treatment (n=54)** | **Routine care (n=36)** | **p-value** |
| --- | --- | --- | --- |
| **Age, mean (SD)** | 52 (16) | 66 (16) | **<0.001** |
| **Female gender, n (%)** | 33 (61) | 22 (61) | 1.0 |
| **Symptom duration <12 weeks n (%)** | 25 (51) | 12 (36) | 0.19 |
| **66-SJC, median (IQR)** | 5 (3-10) | 8 (4-15) | 0.11 |
| **68-TJC, median (IQR)** | 12 (7-19) | 13 (8-19) | 0.69 |
| **ESR, median (IQR)** | 25 (9-43) | 34 (10-62) | 0.21 |
| **DAS44, median (IQR)** | 3.1 (2.6-4.0) | 2.3 (2.6-4.2) | 0.48 |
| **ACPA-positive, n (%)*** | 23 (43) | 3 (8) | **0.001** |
| **RF-positive, n (%)*** | 29 (54) | 9 (25) | **0.007** |

Table depicting baseline characteristics of all patients at the time of inclusion in the EAC-cohort who achieved DMARD-free remission. Missings were as follows: ACPA (1), TJC (2), SJC (1), symptom duration (8)
*ACPA positive if≥7 U/mL), IgM rheumatoid factor (RF) (positive if≥3.5 IU/mL)
RA, rheumatoid arthritis; SD, standard deviation; SJC, swollen joint count; TJC, tender joint count; IQR, interquartile range; ESR, erythrocyte sedimentation; EAC, early arthritis clinic

**Table S2: Univariable Cox-regression analyses studying the association between baseline characteristics and late flares in patients at risk for a late flare**

|  | **No late flare (n=76)** | **Late flare (n=14)** | **HR (95% CI)** | **p-value** |
| --- | --- | --- | --- | --- |
| **Age, mean (SD)** | 58 (18) | 56 (14) | 1.0 (0.97-1.02) | 0.77 |
| **Female gender, n (%)** | 48 (63) | 7 (50) | 0.67 (0.23-1.90) | 0.45 |
| **Symptom duration <12 weeks n (%)** | 37 (49) | 4 (29) | 2.3 (0.7-7.3) | 0.16 |
| **66-SJC, median (IQR)** | 6 (3, 11) | 8 (3, 14) | 1.01 (0.93-1.09) | 0.81 |
| **68-TJC, median (IQR)** | 14 (8, 21) | 12 (8, 12) | 0.95 (0.89-1.02) | 0.14 |
| **ESR, median (IQR)** | 32 (10, 55) | 24 (8, 54) | 1.0 (0.99-1.02) | 0.96 |
| **ACPA-positive, n (%)** | 19 (25) | 7 (50) | 1.23 (1.02-1.50) | **0.034** |
| **RF-positive, n (%)** | 27 (36) | 11 (79) | 6.34 (1.77-22.79) | **0.005** |
| **Intensive treatment, n (%)** | 43 (57) | 11 (79) | 2.31 (0.64-8.30) | 0.20 |

Only patients at risk for a late flare (DMARD-free remission ≥1 year, n=90) were studied. Baseline characteristics of patients with and without a late flare and results of univariable Cox-regression analyses are depicted.
HR, hazard ratio; CI, confidence interval; SD, standard deviation; SJC, swollen joint count; TJC, tender joint count; IQR, interquartile range; ESR, erythrocyte sedimentation rate; ACPA, anticitrullinated protein antibody; RF, rheumatoid factor; HAQ, health assessment

**Figure S1: DAS44 over time**

**
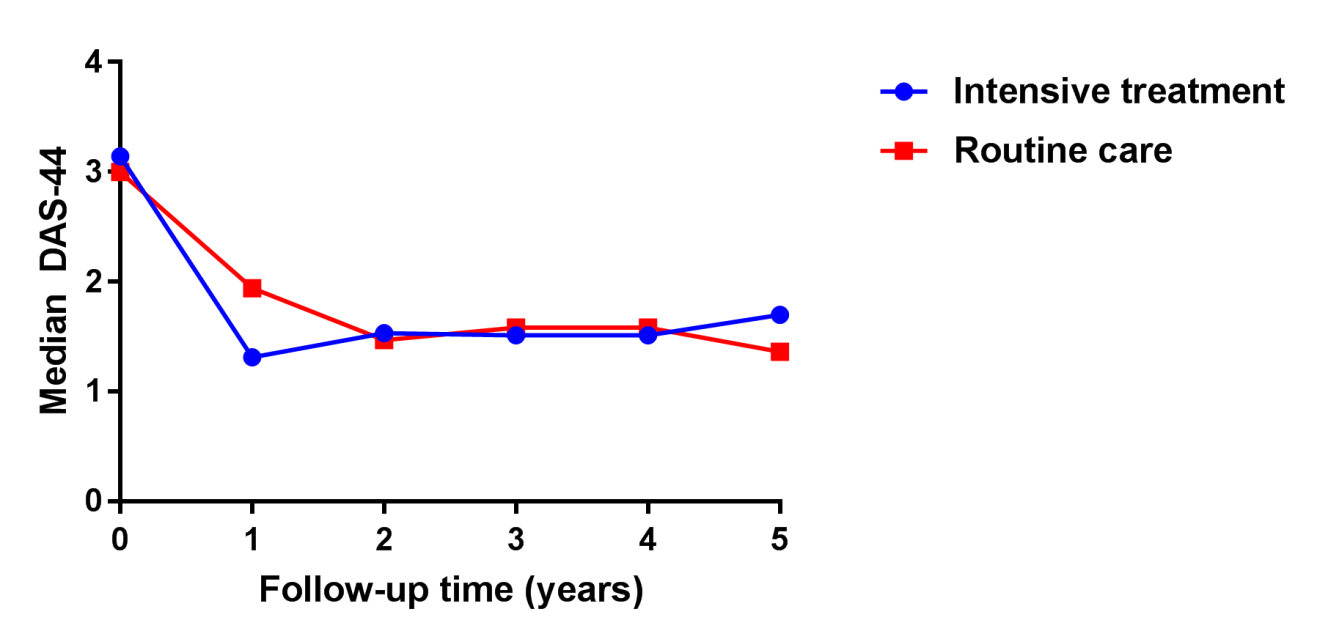
**

DAS, disease activity score
